# Supplementary material for: Causal Relationship between Aging and Anorexia Nervosa: A White-Matter-Microstructure-Mediated Mendelian Randomization Analysis
Source: Biomedicines. 2024 Aug 16;12(8):1874. doi: 10.3390/biomedicines12081874 (PMC11351342; doi:10.3390/biomedicines12081874)
Supplement: Supplementary file 1 [file biomedicines-12-01874-s001.zip › Supplementary Figure/Figure S4/Singe SNP plot of MR analysis for TL to ubm-b-1473.pdf]

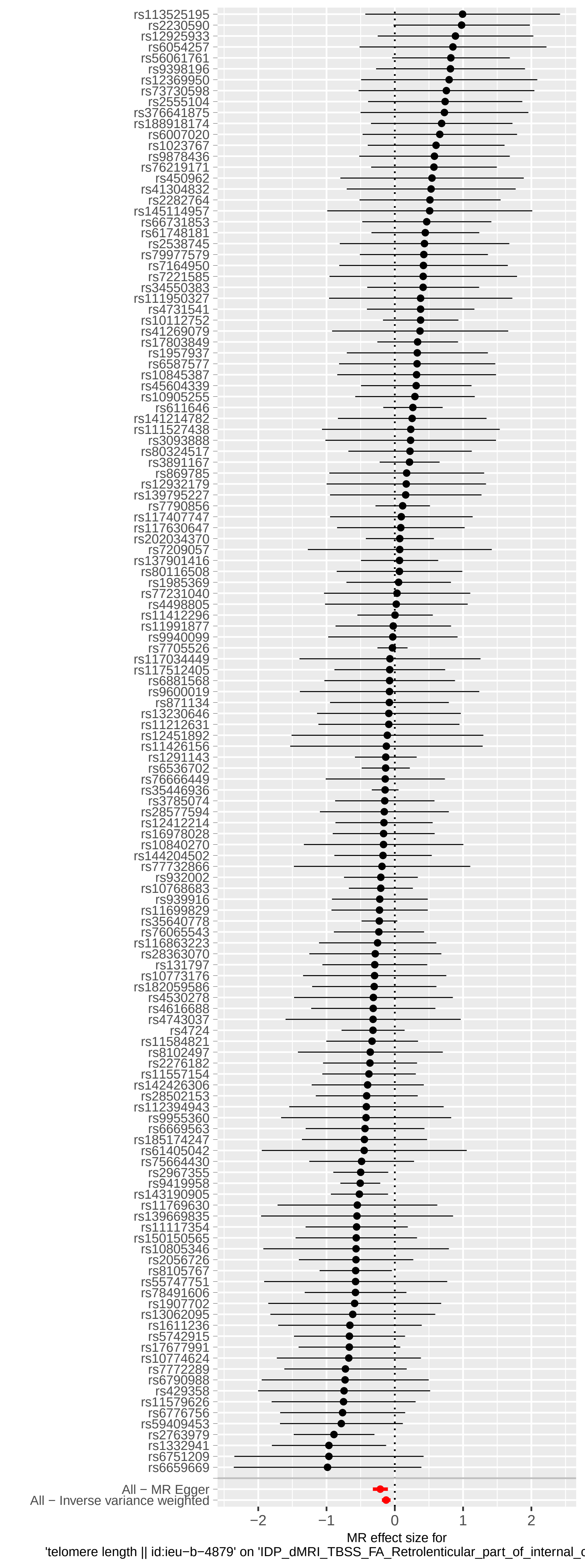
rs113525195
rs2230590
rs12925933
rs6054257
rs56061761
rs9398196
rs12369950
rs73730598
rs2555104
rs376641875
rs188918174
rs6007020
rs1023767
rs9878436
rs76219171
rs450962
rs41304832
rs2282764
rs145114957
rs66731853
rs61748181
rs2538745
rs79977579
rs7164950
rs7221585
rs34550383
rs111950327
rs4731541
rs10112752
rs41269079
rs17803849
rs1957937
rs6587577
rs10845387
rs45604339
rs10905255
rs611646
rs141214782
rs111527438
rs3093888
rs80324517
rs3891167
rs869785
rs12932179
rs139795227
rs7790856
rs117407747
rs117630647
rs202034370
rs7209057
rs137901416
rs80116508
rs1985369
rs77231040
rs4498805
rs11412296
rs11991877
rs9940099
rs7705526
rs117034449
rs117512405
rs6881568
rs9600019
rs871134
rs13230646
rs11212631
rs12451892
rs11426156
rs1291143
rs6536702
rs76666449
rs35446936
rs3785074
rs28577594
rs12412214
rs16978028
rs10840270
rs144204502
rs77732866
rs932002
rs10768683
rs939916
rs11699829
rs35640778
rs76065543
rs116863223
rs28363070
rs131797
rs10773176
rs182059586
rs4530278
rs4616688
rs4743037
rs4724
rs11584821
rs8102497
rs2276182
rs11557154
rs142426306
rs28502153
rs112394943
rs9955360
rs6669563
rs185174247
rs61405042
rs75664430
rs2967355
rs9419958
rs143190905
rs11769630
rs139669835
rs11117354
rs150150565
rs10805346
rs2056726
rs8105767
rs55747751
rs78491606
rs1907702
rs13062095
rs1611236
rs5742915
rs17677991
rs10774624
rs7772289
rs6790988
rs429358
rs11579626
rs6776756
rs59409453
rs2763979
rs1332941
rs6751209
rs6659669
All − MR Egger
All − Inverse variance weighted
−2
−1
0
1
2
MR effect size for
'telomere length || id:ieu−b−4879' on 'IDP_dMRI_TBSS_FA_Retrolenticular_part_of_internal_c
